# Identification, isolation, structural characterization, *in silico* toxicity prediction and *in vitro* cytotoxicity assay of simeprevir acidic and oxidative degradation products†

**DOI:** 10.1039/d0ra09253c

**Published:** 2020-11-24

**Authors:** Rasha M. Ahmed, Marwa A. A. Fayed, Mohammed F. El-Behairy, Inas A. Abdallah

**Affiliations:** Department of Pharmaceutical Chemistry, Faculty of Pharmacy, Misr International University Cairo 11341 Egypt; Department of Pharmacognosy, Faculty of Pharmacy, University of Sadat City Sadat City 32897 Egypt; Department of Organic and Medicinal Chemistry, Faculty of Pharmacy, University of Sadat City Sadat City 32897 Egypt; Department of Analytical Chemistry, Faculty of Pharmacy, University of Sadat City Sadat City 32897 Egypt inas.abdallah@fop.usc.edu.eg

## Abstract

Simeprevir is a new direct-acting antiviral drug used for the treatment of chronic hepatitis C. In this work, a simple, fast and economical chromatographic method was developed for the determination of simeprevir in the presence of its acidic and oxidative degradation products. The stress studies performed herein showed that simeprevir degraded under acidic and oxidative conditions but was stable under thermal and alkaline conditions. Chromatographic separation was achieved on a reversed-phase Eclipse XDB C_18_ column (4.6 × 150 mm, 5 μm). The mobile phase consisted of methanol-0.05 M ammonium acetate (pH 4) (90 : 10, v/v) and was used at a flow rate of 1 mL min^−1^. The column effluent was monitored at 237 nm. The calibration curve was linear over the concentration range of 0.1–20 μg mL^−1^. The relative standard deviations for the intra-day and inter-day precision were less than 2%, and good percentage recoveries that met the acceptance criteria of the International Conference on Harmonization (ICH) guidelines were obtained. The robustness was assessed using the Plackett–Burman design. The simeprevir degradation products were isolated by flash chromatography and confirmed by ^1^H NMR and LC-MS/MS techniques. The fully validated chromatographic method can be applied as a stability-indicating method for simeprevir and for routine analysis during quality control. Additionally, *in silico* toxicity prediction of the degradation products demonstrated a hepatotoxicity alert for DP 1, DP 2, DP 4 and DP 5 and a carcinogenicity alert for DP 3. In view of safety aspects, an *in vitro* cytotoxicity assay was carried out for simeprevir degradation products. They were found to be non-toxic *in vitro* at the tested concentrations.

## Abstract 2

Simeprevir is a new direct-acting antiviral drug used for the treatment of chronic hepatitis C.

## Graphical abstract

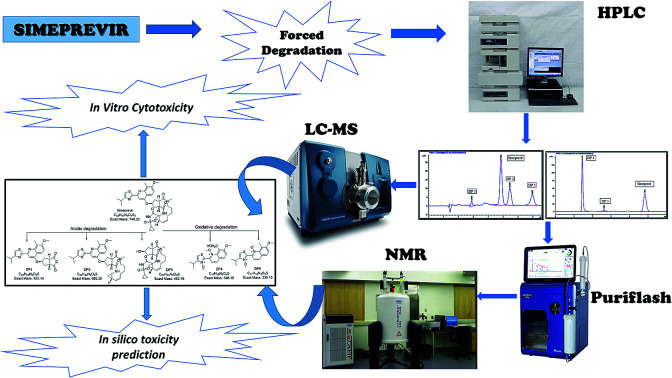

## 1. Introduction

Hepatitis C is a liver disease caused by a small, positive stranded ribonucleic acid virus.^1^ This infectious disease affects approximately 150 million people, and those infected eventually suffer from complications, such as ascites, fibrosis and carcinoma.^2–4^ The emergence of hepatitis C drug treatment has led to a reduction in the number of patients who reach end-stage liver disease and suffer from its complications.

Simeprevir, a second-generation NS3-4A protease inhibitor, is one of the most recent direct-acting antiviral drugs used for the treatment of genotype I hepatitis C.^5^ It is administered in combination with interferon and ribavirin. The mode of cleavage of encoded polyproteins into individual viral proteins.^6–9^

Simeprevir is rapidly absorbed after oral administration and is then metabolized by oxidation by cytochrome P450 in the liver and reaches its maximum plasma concentration between 4 to 6 hours after administration.^10,11^

Quality testing of active pharmaceutical ingredients or pharmaceutical products during their storage and shelf life is crucial.^12^ According to the ICH guidelines, stress studies should be carried out by testing the substance under different conditions, such as acidic, alkaline, oxidative and thermal conditions. Such studies are performed to determine the behaviour of the drug molecule and predict the changes that will occur during storage.^13,14^

A literature survey revealed that different analytical methods for the determination of Simeprevir alone in plasma, including HPLC-DAD^15^ and LC-MS/MS,^16^ or from dosage forms in combination with sofosbuvir by HPLC-DAD,^17^ have been reported. Three stability-indicating methods have been reported for the quantification of simeprevir in the presence of its degradation products, and these methods used HPLC-DAD,^18^ spectrophotometry^19^ and spectrofluorometry.^20^

Although there are several reported analytical methods for the determination of simeprevir, there have been no detailed studies on its degradation products, especially because simeprevir is a non-pharmacopeial drug, and its stability and the identities of its degradation products require further study.

The objective of the present study is to develop an HPLC stability-indicating method to study the degradation behaviour of simeprevir under a variety of degradation conditions, including acidic and oxidative conditions, with high resolution and selectivity. The degradation products of simeprevir will be isolated and characterized then *in silico* toxicity prediction and *in vitro* SRB cytotoxicity assay of all degradation products will be investigated.

## 2. Experimental

### 2.1. Chemicals and reagents

Simeprevir sodium salt was obtained from Hikma Pharmaceutical Industries (Cairo, Egypt). Methanol (HPLC-grade), acetonitrile (HPLC-grade), hydrochloric acid (HCl) and sodium hydroxide (NaOH), deuterated chloroform (CDCl_3_) and deuterated DMSO (DMSO-d_6_) were purchased from Sigma Aldrich (St. Louis, MO, USA). Ammonium acetate, acetic acid and hydrogen peroxide (30% w/v) were purchased from J.T. Baker Chemical Co. (Phillipsburg, NJ, USA). Penicillin–streptomycin (×100), 0.25% trypsin–EDTA, phosphate buffered saline (PBS) and fetal bovine serum (phenol red free) were purchased from Lonza Group Ltd. (Basel, Switzerland). Sulforhodamine-B (SRB), dimethyl sulfoxide (DMSO) and Dulbecco's Modified Eagle Medium (DMEM) were purchased from Sigma-Aldrich (St. Louis, MO, USA). Distilled water was purified by a MilliQ® plus water system (Millipore; Billerica, MA, USA).

### 2.2. Cell culture

Human Skin Fibroblast cell line was obtained from Nawah Scientific Inc., (Mokatam, Cairo, Egypt). Cells were maintained in DMEM media supplemented with 100 mg mL^−1^ of streptomycin, 100 units per mL of penicillin and 10% of heat-inactivated fetal bovine serum in humidified, 5% (v/v) CO_2_ atmosphere at 37 °C.

### 2.3. Instrumentation

Chromatographic separations were carried out on an Agilent 1100 separation system (Agilent Technologies, USA). The high-performance liquid chromatography (HPLC) system was equipped with an online degasser, an isocratic pump and a UV detector.

Isolation of acidic and oxidative degradation products of simeprevir was carried out on Flash chromatography apparatus (puriFlash XS 520 Plus) (Interchim, France).

The HPLC system was controlled by Agilent LC Chemstation 1100 software. The degradation products were structurally characterized using an AB Sciex series LC system (Applied Biosystems Sciex, Ontario, Canada) separation module connected to an API 4500 triple quadrupole mass spectrometer (Applied Biosystems Sciex, Ontario, Canada) coupled with an ion source (Turbo ion spray) that was operated in the positive electrospray ionization (ESI) mode.

The mass spectrometric data were acquired using Analyst 1.6.3 software. The typical operating source conditions for MS scans for detecting simeprevir and its degradation products were optimized to the following: curtain gas (CUR): 20 psi, ion spray voltage (IS): 5500 V, temperature (TEM): 500 °C, declustering potential (DP): 30 V, ion source gas 1 (GS 1): 20 psi, ion source gas 2 (GS 2): 20 psi. Air was used as the nebulizer gas, while nitrogen was used as the auxiliary gas.

The ^1^H experiments were performed on a 400 MHz NMR (ADVANCE III HD-500, Bruker, Fällanden, Switzerland) spectrometer using DMSO-d_6_ and CDCl_3_ as solvent. The chemical shift values are reported on the *δ* scale in ppm relative to TMS (*δ* = 0.00 ppm) as the internal standard.

Absorbance was measured in the cytotoxicity assay at 540 nm on a BMG LABTECH®-FLUOstar Omega microplate reader (Ortenberg, Germany).

### 2.4. Chromatographic conditions

Chromatographic separations were achieved on a Zorbax Eclipse XDB C_18_ column (150 × 4.6 mm, 5 μm) (Agilent Technologies, USA). The mobile phase composition was (A): methanol and (B): 0.01 M ammonium acetate buffer (pH = 4) (A: 90, B: 10, v/v) used at a flow rate of 1 mL min^−1^.

A detection wavelength of 237 nm and an injection volume of 20 μL were selected for the determination of Simeprevir and its degradation products. All chromatograms were acquired at room temperature.

### 2.5. Forced degradation study

The forced degradation studies were carried out on 1 mg mL^−1^ in methanol. Acidic hydrolysis was performed by treating the drug with 0.5 M hydrochloric acid for 5 hours, while the basic hydrolysis conditions were 1 M sodium hydroxide for 5 hours. In both cases, the reaction was immersed in a boiling water bath maintained at 100 °C.

Oxidative degradation was performed by treating the drug with 3% hydrogen peroxide for 5 hours and the reaction was immersed in a boiling water bath maintained at 100 °C. In the thermal degradation, simeprevir was subjected to high temperature by placing the drug in a thermostatic oven at 100 °C.

### 2.6. Isolation of degradation products by flash chromatography

A Flash chromatography apparatus (puriFlash XS 520 Plus) was used for purification of simeprevir acidic and oxidative degradation products using normal phase silica gel column (12 g × 30 μm) and hexane/methylene chloride mobile phase.

The acidic degradation products mixture consists of the drug and three degradation products according to the recorded HPLC chromatogram was subjected to flash chromatography with an isocratic mobile phase composed of hexane and methylene chloride (1 : 1 v/v). The flow rate was set at 15 mL min^−1^, where the first acidic degradation product (DP 1) was isolated in pure form. The other two acidic degradation products in addition to the drug that were eluted in several fractions in the form of a mixture which were monitored using thin layer chromatography (TLC) and similar fractions were gathered and combined depending on the number and color of the spots under UV light. Then the mixture was concentrated and reloaded on silica gel column using flash chromatography under the same previous conditions and with a gradient of hexane/methylene chloride used as the mobile phase, starting with 100% hexane and increasing polarity till 100% methylene chloride where the two acidic degradation products (DP 3, DP 2) were isolated single and pure.

Purification of the oxidative degradation products mixture, which consists of the drug with two degradation products (DP 5, DP 4) according to the recorded HPLC chromatogram, was carried out under the same conditions of flash chromatography and hexane/methylene chloride gradient as mobile phase starting with hexane (100%) then increasing the polarity till (100%) methylene chloride where both were isolated in pure form.

Both acidic degradation products (DP 1, DP 2 and DP 3) and oxidative degradation products (DP 4 and DP 5) were obtained as pure solids after concentration and removal of the mobile phase.

### 2.7. Preparation of the samples for chromatographic analyses

The samples from the acidic and basic hydrolysis stress tests were neutralized and diluted with methanol to achieve final assay concentrations of 100 μg mL^−1^. The final sample solutions were filtered through a 0.45 μm membrane before HPLC analysis. All sample solutions were stored at 4 °C in a refrigerator to avoid degradation.

### 2.8. Method validation

The proposed chromatographic method was validated with respect to selectivity, limit of detection (LOD), limit of quantification (LOQ), linearity, accuracy, precision and robustness as described by the ICH guidelines Q2 (R1).^21^

### 2.9. Application of the developed HPLC method

The developed stability-indicating assay was applied to the analysis of simeprevir in a commercial formulation. A standard addition technique was used to assess the developed and validated HPLC method.

### 2.10. *In silico* toxicity studies

The ADMET descriptors and toxicity parameters of the tested compounds were calculated using Discovery studio 4.0. At first, the CHARMM force field was applied then the compounds were prepared and minimized according to the preparation of small molecule protocol. Then different parameters were calculated from toxicity prediction (extensible) protocol. Also, the ADMET descriptors protocol was applied to carry out these studies.^22,23^

2D structures of the tested compounds were sketched using ChemBioDraw Ultra 14.0 and saved in MDL-SD file format. SD file was opened, 3D structures are protonated and energy minimized by applying CHARMM force fields for charge, and MMFF94 force field for partial charge. Then, the tested compounds were prepared using prepare ligand option. In which, we used rule based option for ionization mode. At the same time, the tautomer generation, isomer generation, and fixation of bad valences were adjusted to be false. Next, the prepared compounds were subjected to toxicity calculation process using toxicity prediction (extensible) option. In which, the models panel was feed by different model names. In addition, the similarity search was adjusted to be true. The force field based scoring functions (CHARMM force fields for charge, and MMFF94 force field for partial charge) were used in this study.^24^

### 2.11. Cytotoxicity assay

Cell viability was assessed by SRB assay.^25^ Aliquots of 100 μL cell suspension (5 × 10^3^ cells) were in 96-well plates and incubated in complete media for 24 h. Cells were treated with another aliquot of 100 μL media containing (DP 1, DP 2, DP 3, DP 4, DP 5 and Doxorubicin as positive control) at various concentrations ranging from (0.01, 0.1, 1, 10, 100 μm). After 72 h of drug exposure, cells were fixed by replacing media with 150 μL of 10% TCA and incubated at 4 °C for 1 h. The TCA solution was removed, and the cells were washed 5 times with distilled water. Aliquots of 70 μL SRB solution (0.4% w/v) were added and incubated in a dark place at room temperature for 10 min. Plates were washed 3 times with 1% acetic acid and allowed to air-dry overnight. Then, 150 μL of Tris (10 mM) was added to dissolve protein-bound SRB stain; the absorbance was measured at 540 nm using microplate reader. The percentage of cell viability was calculated by using the following formula:^26^
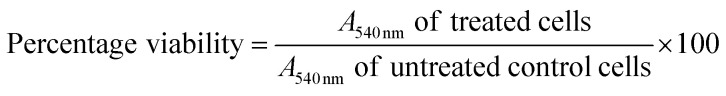


## 3. Results and discussion

### 3.1. HPLC method development and optimization

The chromatographic method was developed by selecting a mobile phase and stationary phase suitable for the separation of simeprevir from its degradation products. Preliminary studies were performed using a C_18_ column (150 × 4.6 mm, 5 μm) and trying different mobile phases with different polarities to identify chromatographic conditions well suited to the structural features of simeprevir. A mobile phase with a high percentage of an organic modifier was selected and using water as the aqueous phase was not suitable because the peak shape and symmetry were poor. Subsequently, ammonium acetate buffers at different concentrations (0.01–0.05 M) and different pH values (3–5) were screened to improve the shape and symmetry of the peaks. Peak tailing was observed upon using highly concentrated acetate buffers, while the pH 4 buffer worked well because the pH was well away from the p*K*_a_ of simeprevir (p*K*_a_ = 5.9). Detection of simeprevir was performed at 237 nm which was observed as wavelength of maximum absorbance (*λ*_max_) after UV scanning of drug solution as shown in Fig. S1.†

**Fig. 1:**
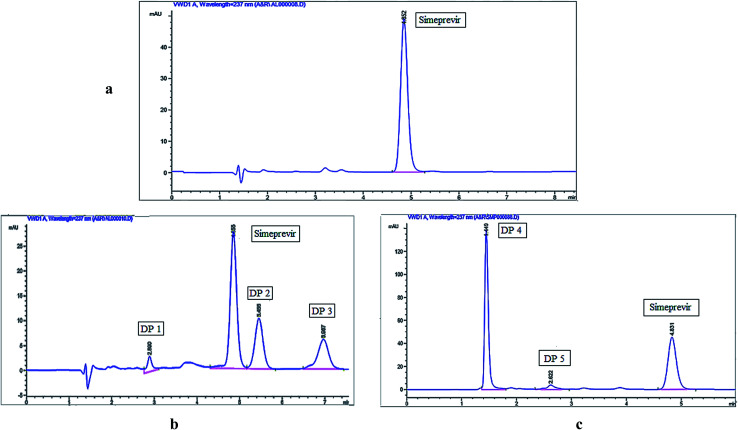
HPLC chromatograms of (a) simeprevir (10 μg mL^−1^), (b) acidic degradation and (c) oxidative degradation.

Desirable resolution between Simeprevir and its degradation products and acceptable peak shapes were observed using isocratic elution as described under chromatographic conditions, and the chromatograms shown in Fig. 1 were acquired using the optimized conditions for the separation of simeprevir from its acidic and oxidative degradation products. Results of system suitability parameters for simeprevir in presence of its degradation products are shown in Table 1.

**Table 1:** System suitability parameters for simeprevir in the presence of its acidic and oxidative DPs

Parameter	Acidic DPs	Oxidative DPs
Resolution	1.75	9.0
Selectivity	1.14	3.0
No. of theoretical plates	5066	5080
Capacity factor	2.2	2.2
Symmetry	0.82	0.81

### 3.2. Forced degradation study

The degradation behaviour of simeprevir was studied according to the ICH guidelines, and the drug was found to be unstable to acidic and oxidative conditions and stable under alkaline and thermal degradation conditions, as shown in Table 2. Although, previous reports by Attia *et al.*^18^ and Mohammed *et al.*^27^ uses different basic hydrolysis conditions; 0.1 N NaOH for 3 hours at room temperature and 1 M NaOH for 2 hours at 60 °C; respectively. Both results were found to be similar where one degradation product was observed with almost same degradation percentage (35.55% and 39.80%). In our report, no peak was observed after HPLC injection of the alkaline degradation samples which is confirmed using TLC and Flash Chromatography; in which only drug peak was detected.

**Table 2:** Summary of stress degradation of simeprevir^a^

Degradation study	Exposure conditions	Time	DPs formed & *R*_t_ (min)	% Degradation
Acidic degradation	0.5 M HCl at 100 °C	5 hours	DP 1 (*R*_t_ = 2.890)	26.63
			DP 2 (*R*_t_ = 5.455)	
			DP 3 (*R*_t_ = 6.987)	
Alkaline degradation	1 M NaOH at 100 °C	5 hours	No DPs formed	No degradation
Oxidative degradation	3% w/v H_2_O_2_ at 100 °C	5 hours	DP 4 (*R*_t_ = 1.449)	21.87
			DP 5 (*R*_t_ = 2.622)	
Thermal degradation	Oven at 100 °C	10 hours	No DPs formed	No degradation

a DPs: degradation products, *R*_t_: retention time.

All degradation samples were either neutralized as in case of acidic or alkaline hydrolysis or left for residual hydrogen peroxide to be bubbled out. Then all sample were diluted with mobile phase before HPLC injections. All these steps confirms that the degradation products, which were separated on the HPLC chromatograms, were actual degradants and not residual reagents peaks. In addition, the Flash Chromatography step followed by TLC in which we separated degradation products as single compounds confirmed their presence in the sample.

Three degradation products (DP 1, DP 2, and DP 3) were observed using HPLC-UV when simeprevir was subjected to acidic hydrolysis, while two degradation products (DP 4 & DP 5) were obtained under oxidative degradation. Simeprevir was more susceptible to acidic degradation than to oxidative degradation, as 26.63% degradation was observed under acidic conditions compared to 21.87% under oxidative conditions.

### 3.3. Characterization of degradation products

All degradation products and their fragmentation pattern has been depicted in Fig. 3–5.

As per (Fig. S2†), the ^1^H-NMR of intact Simeprevir drug has shown the characteristic 4 aromatic protons, one proton at *δ* 8.03 ppm (1H) of thiazole rings plus three at *δ* 7.52–7.39 ppm (3H) of quinoline ring. Also, amide protons were recognized as 2 singlet signals at *δ* 7.26 ppm (1H) and 5.33 ppm (1H). The alkene protons in the macrocycle were detected at *δ* 5.45 ppm (2H). The most de-shielded proton of cyclopentane ring attached to oxygen at position 4 of quinoline ring was spotted at *δ* 4.34–4.20 ppm (1H) as multiplet signal due to coupling with adjacent protons of the cyclopentane ring. The singlet signal corresponding to methoxy group at 7 position of quinoline ring was at *δ* 3.96 ppm (s, 3H) while the singlet signal corresponding to *N*-methyl group was at *δ* 3.09 ppm (s, 3H). The methyl group at position 8 of quinoline ring was noticed as singlet signal at *δ* 2.58 ppm (s, 3H). Further aliphatic protons of isopropyl group (6H) on thiazole and cyclopropyl group (4H) attached to sulfur was detected at *δ* 1.34 ppm (6H), *δ* 0.78 ppm (2H), and 0.62 ppm (2H) respectively.

#### 3.3.1. Characterization of DP 1

**Fig. 2:**
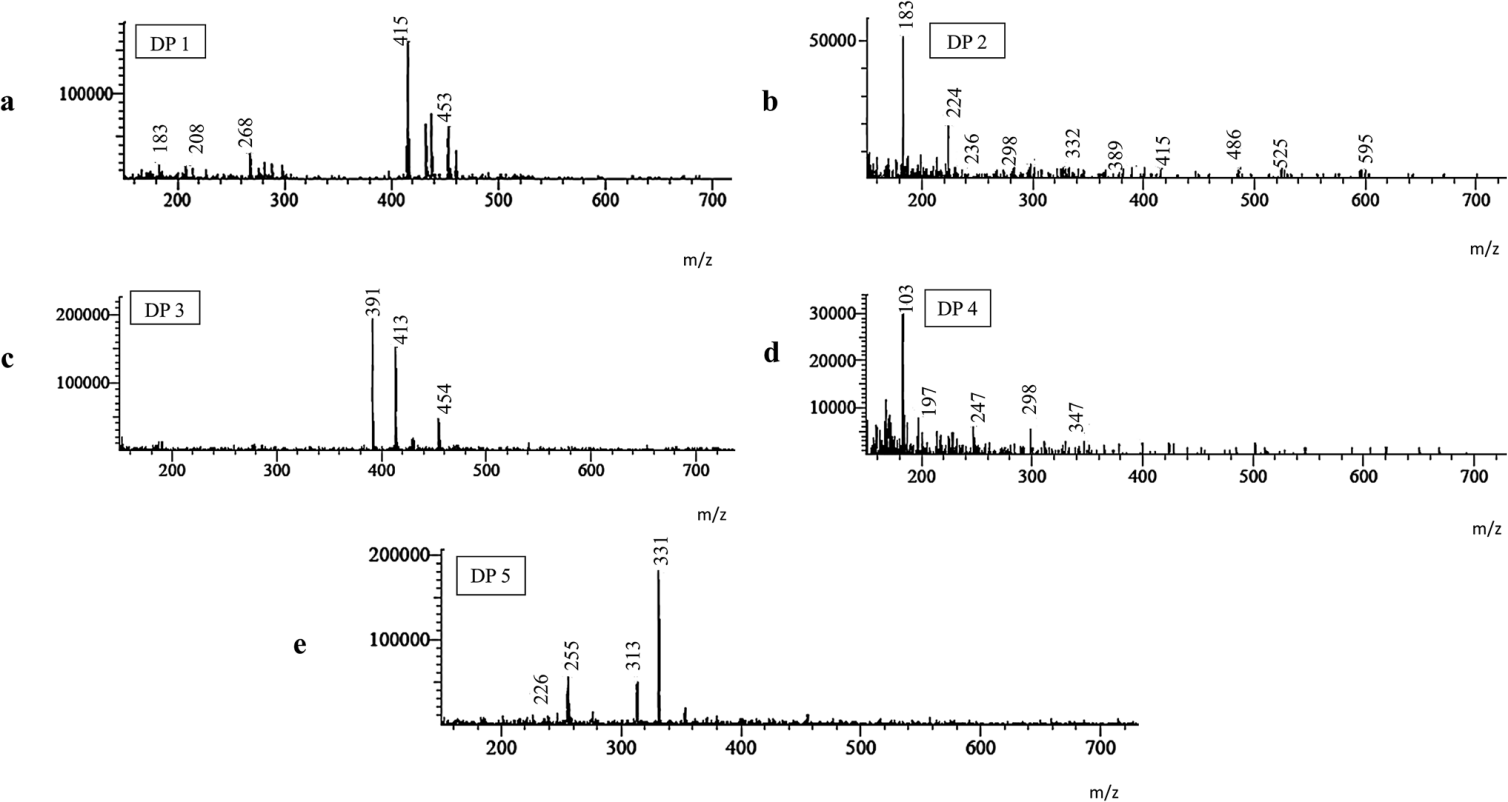
Mass spectra of degradation products: (a) DP 1, (b) DP 2, (c) DP 3, (d) DP 4 and (e) DP 5.

**Fig. 3:**
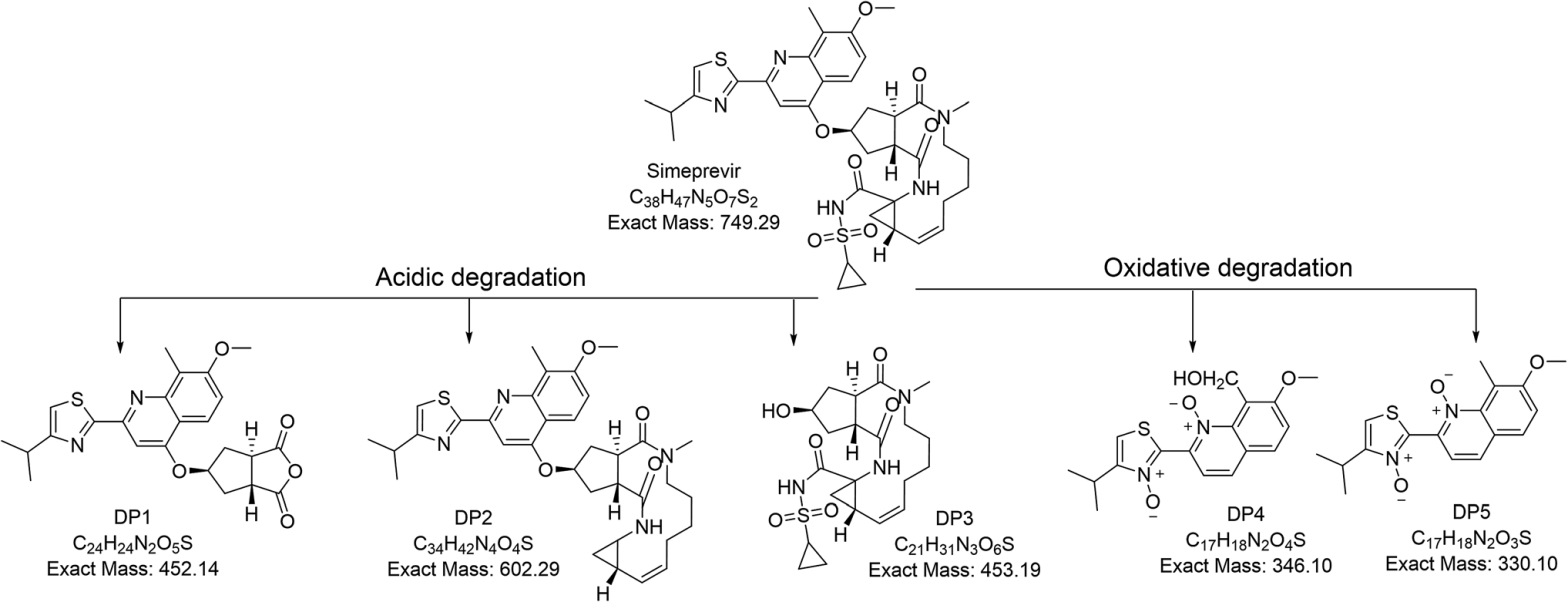
Degradation pathways of simeprevir.

**Fig. 4:**
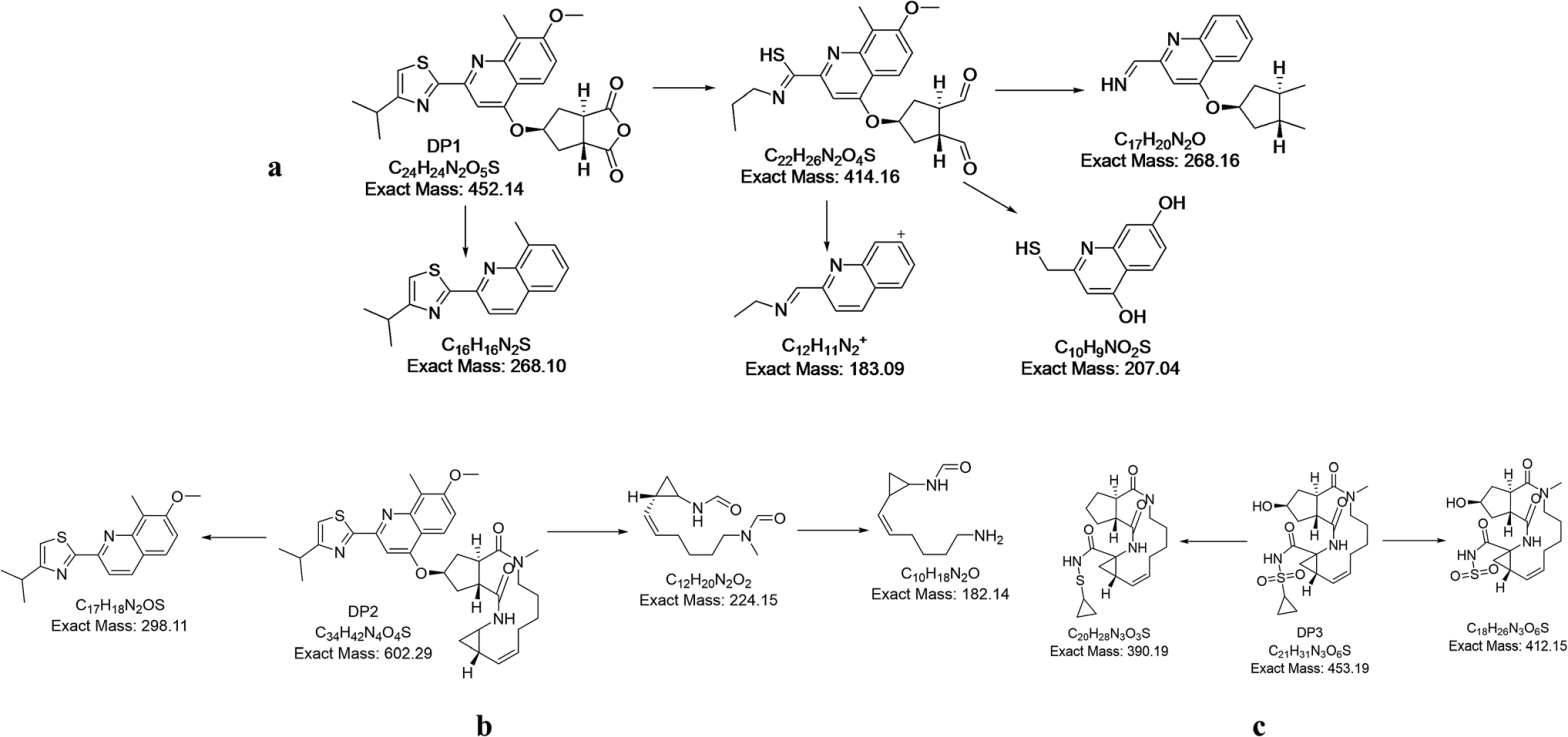
Fragmentation pathway of (a) DP 1, (b) DP 2 and (c) DP 3.

Mass spectrum of DP 1 (Fig. 2) has shown molecular ion peak at 453 *m*/*z* which is corresponding to [M + 1]^+^ of the depicted acidic degradation product 1 (DP 1) in Fig. 3. Also, its expected fragmentation pattern has been portrayed in Fig. 4. The structure of DP 1 (Fig. 3) has been confirmed by ^1^H-NMR (Fig. S3†) where both amidic protons have disappeared thus all amide bonds are expected to be broken. This has been supported by the loss of the upfield signals of cyclopropyl protons of cyclopropylacylsulfonamide moiety that was noticed at *δ* 0.78 ppm (2H), 0.62 ppm (2H). Also, alkene and the *N*-methyl protons in the macrocycle were undetectable. On the other hand, four aromatic protons, the singlet signals corresponding to methoxy group at position 7 and methyl group at position 8 of quinoline ring, and the aliphatic protons of isopropyl group (6H) on thiazole ring were still noticed. Such observations suggests the breakage of both amide bonds in the macrocycle.

#### 3.3.2. Characterization of DP 2

For acidic degradation product 2 (DP 2) (Fig. 3), mass spectrum has displayed the molecular ion peak at 602 *m*/*z* which is corresponding to [M + 1]^+^ of the depicted acidic degradation product 2 at (Fig. 2) and its fragmentation pattern (Fig. 4). Further confirmation of the structure *via*^1^H-NMR (Fig. S4†) has been performed where one amidic protons have disappeared. This has been supported by the loss of the upfield signals of cyclopropyl protons of cyclopropylacylsulfonamide moiety that was noticed at *δ* 0.78 ppm (2H), 0.62 ppm (2H) and the amidic proton at *δ* 5.33 ppm in simeprevir spectrum. On contrary to DP 1, alkene protons at *δ* 5.37 ppm (2H) and the *N*-methyl protons at *δ* 3.68 ppm in the macrocycle were detectable. Also, four aromatic protons and one amidic proton at 7.73–7.10 ppm were spotted. The most de-shielded proton of cyclopentane ring that attached to oxygen at position 4 of quinoline ring and the signal corresponding to methoxy group at position 7 were still noticed at *δ* 4.39–4.13 ppm (4H). Such observations suggests the breakage of the bond between the cyclopropylacylsulfonamide moiety and the macrocycle.

#### 3.3.3. Characterization of DP 3

For acidic degradation product 3 (DP 3) (Fig. 3), mass spectrum (Fig. 2) has displayed the molecular ion peak at 454 *m*/*z* which is corresponding to [M + 1]^+^ of the depicted acidic degradation product 3 (Fig. 2) and its fragmentation pattern (Fig. 4). Further confirmation of the structure *via*^1^H-NMR (Fig. S5†) has been performed and revealed the disappearance of all aromatic protons. While two amidic protons at *δ* 7.74–7.54 ppm and alkene protons at *δ* 5.37 ppm (2H) were still noticed. Such observations suggests the breakage of the bond between the macrocycle and the aromatic moiety to afford degradant 3.

#### 3.3.4. Characterization of DP 4 and DP 5

**Fig. 5:**
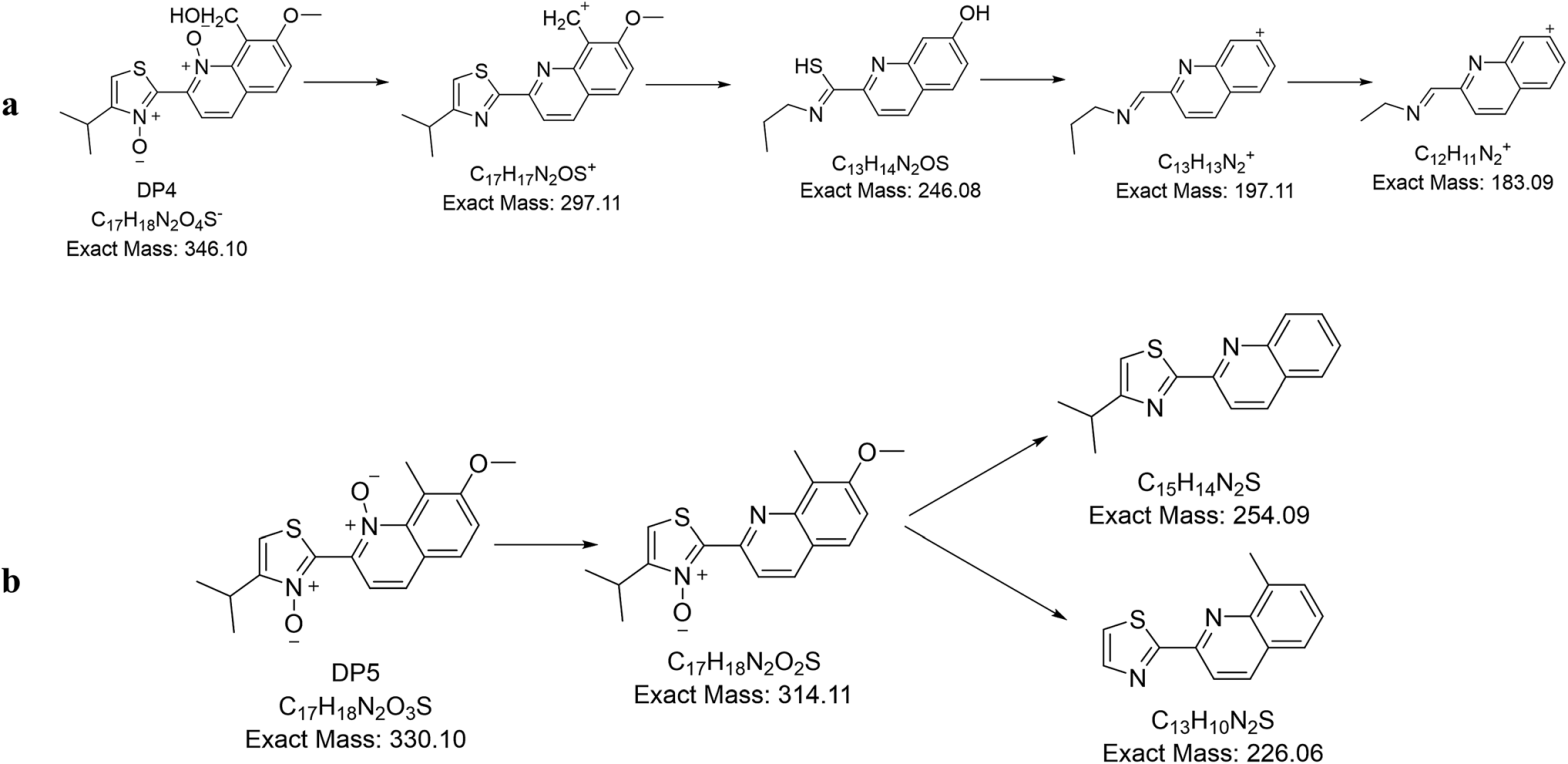
Fragmentation pathway of (a) DP 4 and (b) DP 5.

Oxidative degradation is expected to produce N-oxide from tertiary amines (but not from amide)^28^ and aromatic nitrogens while NH and NH_2_ are expected to afford N–OH and NO_2_ respectively.^28,29^ In the oxidative degradation of simeprevir, two oxidative degradants have been isolated where mass spectroscopy showed *m*/*z* 347 and 331 that corresponding to [M + 1]^+^ of degradants DP 4 and DP 5 respectively (Fig. 2) and their fragmentation patterns (Fig. 5). 331 *m*/*z* is corresponding to degradant with both nitrogens of quinolone and thiazole were converted to the N-oxide. While 347 *m*/*z* is for the alcohol produced by the oxidation of the methyl group at position 8 of quinoline ring (Fig. 3). As a result, ^1^H-NMR (Fig. S6 & S7†) of both degradants were almost identical except for the signal at *δ* 5.32 ppm which showed the doublet of doublet manner (looks like multiplet) of prochiral carbon where protons are showing nonequivalent shift and couples to each other.^30^ Postulated mechanisms of degradation is illustrated in Fig. S8 & S9.†

### 3.4. Method validation

#### 3.4.1. Linearity

Solutions at six different concentrations were prepared from the standard solution of simeprevir, and a calibration curve was constructed by plotting the peak areas as a function of concentration. The regression equation was calculated to be *Y* = 54.75*X* + 15.28, and the correlation coefficient was found to be 0.9997, as shown in Table 3, indicating satisfactory linearity in the proposed method.

**Table 3:** Summary of validation parameters of chromatographic method used for determination of simeprevir

Parameter	Simeprevir
**Linearity**	
Regression equation	*Y* = 54.75*X* + 15.28
Range (μg mL^−1^)	0.1–20
Correlation coefficient (*r*)	0.9997
Slope	54.75
Intercept	15.28
LOD (μg mL^−1^)	0.10
LOQ (μg mL^−1^)	0.34

**Precision**	
*Repeatability (intra-day) (% RSD)* a	
QCL (0.5 μg mL^−1^)	1.80%
QCM (7 μg mL^−1^)	1.03%
QCH (15 μg mL^−1^)	0.71%
*Intermediate precision (inter-day) (% RSD)* a	
QCL (0.5 μg mL^−1^)	0.70%
QCM (7 μg mL^−1^)	0.44%
QCH (15 μg mL^−1^)	0.57%

**Accuracy**	
*(Mean ± S.D)* b	
QCL (0.5 μg mL^−1^)	100.80 ± 1.54
QCM (7 μg mL^−1^)	99.43 ± 1.34
QCH (15 μg mL^−1^)	100.67 ± 0.63

a RSD: relative standard deviation.

b Expressed mean of three replicates.

#### 3.4.2. Precision

The repeatability (intra-day) and intermediate precision (inter-day) were assessed by analysing solutions of three different concentrations on the same day and the three sequential days, respectively. The relative standard deviations were below 2% (Table 3). Therefore, the method was considered sufficiently precise.

#### 3.4.3. Accuracy

The accuracy of the method was determined by spiking simeprevir into synthetic mixtures with the degradation products at three different concentrations (0.5, 7 and 15 μg mL^−1^) in triplicate. Then calculating the percentage recoveries of these three different concentrations of simeprevir (Table 3). Acceptable percentage recoveries were obtained (between 99% and 101%). Hence, the developed stability-indicating method is accurate.

#### 3.4.4. Specificity

The specificity was examined by analysing three laboratory-prepared mixtures of the acidic and oxidative degradation products with known concentrations of the intact drug within the linear region. There was no interference between the degradation products and intact simeprevir.

#### 3.4.5. Detection limit (LOD) & quantitation limit (LOQ)

The LOD and LOQ were investigated and found to be 0.1 and 0.34 μg mL^−1^, respectively (Table 3).

#### 3.4.6. Robustness

Plackett–Burman design was used to assess the robustness of the method. Small deviations from the method conditions were examined, and corresponding responses were observed. Eleven randomized runs including three centre points were performed under different conditions as presented in Table 4. All parameters were found to be non-significant at *p* values below 0.5, and the coefficient plot (Fig. S10†) confirmed the non-significances of these parameters concerning the resolution of simeprevir with its degradation products, the theoretical plates and selectivity of Simeprevir.

**Table 4:** Design of experiment (DOE) for simeprevir robustness testing

Exp. no.	pH	Methanol	Wavelength	Flow rate
1	4.2	88	235	1.2
2	4.2	92	235	0.8
3	4.2	92	239	0.8
4	3.8	92	239	1.2
5	4.2	88	239	1.2
6	3.8	92	235	1.2
7	3.8	88	239	0.8
8	3.8	88	235	0.8
**9**	**4**	**90**	**237**	**1**
**10**	**4**	**90**	**237**	**1**
**11**	**4**	**90**	**237**	**1**

### 3.5. Application to pharmaceutical dosage form

The validated method was successfully applied for the determination of simeprevir in its dosage form, and satisfactory results were obtained with a good recovery (101.34%). A standard addition technique was used (Table 5) to verify the method, and the study revealed no interference from the excipients.

**Table 5:** Determination of simeprevir in its dosage form and application of standard addition technique

	Claimed taken (μg mL^−1^)	Found (%)	Pure added (μg mL^−1^)	Recovery%
Merospevir® hard gelatin capsules (B.N. 160117)	7	101.34 ± 1.66	3	101.71 ± 0.93
			7	101.56 ± 0.13
			8	100.85 ± 0.46

### 3.6. *In silico* toxicity studies

Toxicity prediction was carried out for our degradation products based on the validated and constructed models in Discovery studio software.^31,32^ The toxicity of the tested compounds against central nervous system and liver have been determined in ADMET study.

ADMET – Blood Brain Barrier (BBB) penetration studies predicted that BBB penetration levels of DP 2 and DP 3 are very low. While DP 4 exhibited low level and DP 1 and DP 5 showed medium BBB penetration levels. Accordingly, all compounds were expected to be safe to CNS. The hepatotoxicity model predicts potential organ toxicity for a wide range of structurally diverse compounds.^33^ All examined compounds were demonstrated to have some a sort of hepatotoxicity except DP 3. Further *in vitro* and *in vivo* are required to validate this primary *in silico* results.

The measurement of carcinogenic potency for degradation products is an essential factor to understand its risk assessment.^34^ Consequently, three different *in silico* studies have been proceeded as follow; (i) TOPKAT_mouse_male_FDA_none_vs_carcinogen model that is one of FDA rodent carcinogenicity models. The chosen model computes the probability of a submitted chemical structure being a carcinogen or not.^35^ (ii) Carcinogenic potency (TD_50_) which predicts the median tumorigenic dose (the dose required to produce a tumorigenic effect in 50% of rats) of a chemical in a chronic exposure toxicity test.^35^ TD_50_ has been used historically as a metric to determine carcinogenic potency and was included in the Carcinogenic Potency Data Base (CPDB)^36^ and (iii) developmental toxicity potential which predicts whether a chemical compound is likely to be toxic in a developmental toxicity potential assessment. The developmental toxicity is any functional or structural change, either reversible or irreversible, that interferes and alter homeostasis, normal growth, differentiation, development or behaviour.^37,38^

On the other hand, to reveal the acute and chronic toxicity of the examined compounds, three other *in silico* experiments were done (i) rat maximum tolerated dose (MTD) which predicts the highest dose of a chemical that expected to produce the desired effect without causing unacceptable side effects.^39,40^ (ii) Rat oral LD_50_ which predicts the rat oral acute median lethal dose (LD_50_) in the toxicity test of a chemical^41^ and (iii) rat chronic (LOAEL) which predicts the rat chronic lowest observed adverse effect level (LOAEL) value of a chemical.^42^

As shown in Table 6, most compounds showed *in silico* very low adverse effect and toxicity against the tested models. For FDA Rodent Carcinogenicity model, all compounds were appeared to be non-carcinogen except DP 3. For carcinogenic potency TD_50_ mouse model, compounds DP 1 and DP 2 showed low TD_50_ values, while DP 2, DP 4, and DP 5 showed high TD_50_ values. Regarding rat maximum tolerated dose model, the examined compounds showed maximum tolerated dose with a range of 0.006 to 0.020 g kg^−1^ body weight. Additionally, all compounds were non-toxic against developmental toxicity potential model. For rat oral LD_50_ model, all compounds showed low oral LD_50_ values ranging from 0.080 to 0.352 mg per kg body weight per day. For rat chronic LOAEL model, the compounds showed LOAEL with a range of 0.005 to 0.023 g kg^−1^ body weight.

**Table 6:** *In Silico* toxicity studies of simeprevir degradation products

	DP 1	DP 2	DP 3	DP 4	DP 5
TOPKAT_mouse_male_FDA_none_vs_carcinogen model	Non-carcinogen	Non-carcinogen	Carcinogen	Non-carcinogen	Non-carcinogen
Carcinogenic potency TD_50_ mouse^a^	11.613	3.910	33.345	47.454	73.882
Developmental toxicity potential	Toxic	Non-toxic	Non-toxic	Non-toxic	Non-toxic
Rat maximum tolerated dose^b^	0.018	0.006	0.012	0.020	0.013
Rat oral LD_50_^b^	0.300	0.352	0.290	0.115	0.080
Rat chronic LOAEL^b^	0.011	0.005	0.005	0.019	0.023
BBB level^c^	2	4	4	3	2
Hepatotoxic prediction^d^	TRUE	TRUE	FALSE	TRUE	TRUE

a Unit: mg per kg body weight per day.

b Unit: g kg^−1^ body weight.

c BBB level, blood brain barrier level, 0 = very high, 1 = high, 2 = medium, 3 = low, 4 = very low.

d Hepatotoxicity probability, TRUE means toxic, FALSE means non-toxic.

Assessment of real accelerated stability studies for simeprevir with *in vivo* toxicity prediction of these degradation products are of great importance and will be presented in the near future in a separate study by our research group.

### 3.7. Cytotoxicity assay

Cytotoxicity assay was carried out to confirm the *in silico* toxicity prediction study results. As an *in vitro* method to monitor the potential toxicity and/or safety of simeprevir and its degradation products (DP 1, DP 2, DP 3, DP 4 and DP 5), against Human Skin Fibroblast (HSF) cell line. SRB assay was used to assess the effect of these compounds on the viability and proliferation of HSF cell line (Table S1†), in addition to taking images using the optical microscope to register any change on the tested cells.

The percentage of cell viability was measured at five different concentrations (0.01, 0.1, 1, 10 and 100 μM) in comparison with doxorubicin as a standard drug (positive control) which is monitored at the same concentration range after incubation for 72 h period.

**Fig. 6:**
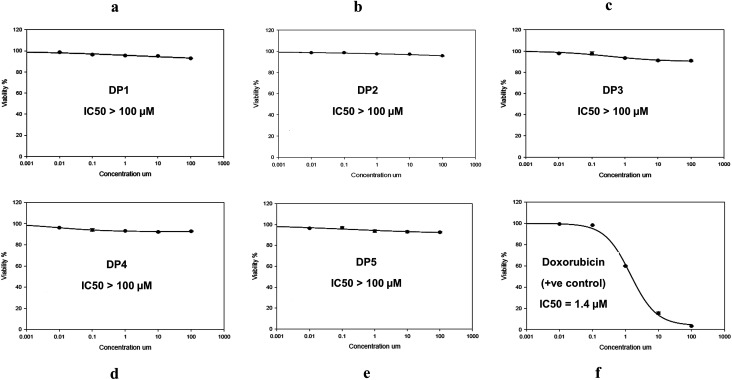
*In vitro* cytotoxicity of (a) DP 1, (b) DP 2, (c) DP 3, (d) DP 4, (e) DP 5 and (f) doxorubicin in increasing concentrations (0.01–100 μM) incubated in Human Skin Fibroblast cell lines (HSF) using SRB viability assay. Data points are expressed as mean ± SD (*n* = 3).

The results of the SRB assay showed excellent correlation with the *in silico* results where no alterations on the cell viability were observed (100.58% ± 0.109) after incubation of the degradation products with HSF cell line as shown in Fig. 6 and Table S1.† As for DP 3, it showed slight cytotoxicity that reached 89.86% in one of the replicates at concentration 100 μM which was suggested as a carcinogenic agent in the *in silico* study. The cell viability range reached 97.91 ± 0.645 and 96.73 ± 0.420 at the same concentration for the other degradation products (DP 1, DP 2, DP 4 and DP 5).

**Fig. 7:**
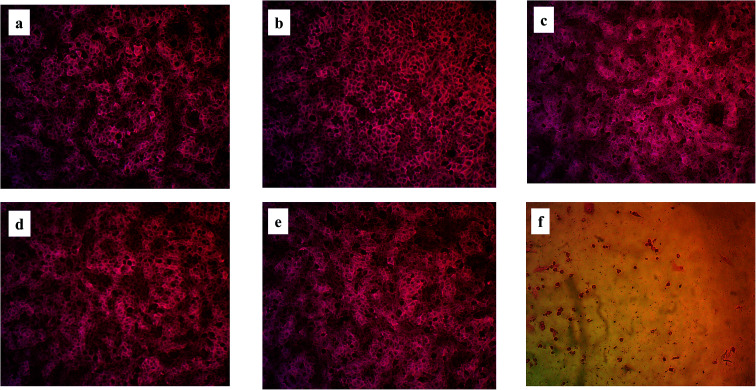
Optical microscope stained images of cytotoxicity assays at HSF cell line (a) DP 1, (b) DP 2, (c) DP 3, (d) DP 4, (e) DP 5 and (f) doxorubicin. All at concentration of 100 μM and magnification power: 200×.

The optical microscope stained images were recorded as shown in Fig. 7 comparing the cytotoxic effect of the five degradation products with that of doxorubicin at a concentration of 100 μM. It shows clearly that no morphological changes occur in case of the degradation products with exception of slight changes in case of DP 3. This proves that simeprevir acidic and oxidative degradation products are non-toxic up to 100 μM (IC_50_ > 100 μM). Same cytotoxic effect comparison was performed between the degradation products and doxorubicin at a lower concentration (0.1 μM) as shown in Fig. S11† with no occurrence of morphological changes in both degradation products and doxorubicin.

## 4. Conclusion

A stability-indicating method for simeprevir was developed according to the ICH guidelines. Simeprevir was found to be susceptible to acidic and oxidative stress and stable under alkaline and thermal stress conditions. Three major degradation products (DP 1, DP 2 and DP 3) from the acidic conditions and two degradation products (DP 4 and DP 5) from the oxidative stress conditions were detected using HPLC-UV. The degradation products were successfully isolated and characterized using LC-MS MS and ^1^H NMR techniques. The proposed acidic and oxidative degradation pathways of simeprevir were outlined and explained. The chromatographic method was validated and found to be accurate, precise, and suitable for application in routine quality control. The *in silico* toxicity prediction revealed the carcinogenic potential of DP 3. All degradation products were found to hepatotoxic except for DP 3 based on high probability scores. *In vitro* cytotoxicity study for the isolated DPs were carried out on Human Skin Fibroblast cell line. All DPs exhibited no toxicity with an IC_50_ > 100 μM.

## Conflicts of interest

There are no conflicts to declare.

## Supplementary Material

RA-010-D0RA09253C-s001
