# Supplementary material for: Identification, isolation, structural characterization, in silico toxicity prediction and in vitro cytotoxicity assay of simeprevir acidic and oxidative degradation products
Source: RSC Adv. 2020 Nov 24;10(70):42816–26. doi: 10.1039/d0ra09253c (PMC9057948; doi:10.1039/d0ra09253c)
Supplement: RA-010-D0RA09253C-s001 [file RA-010-D0RA09253C-s001.pdf]

Electronic Supplementary Material (ESI) for RSC Advances.
This journal is © The Royal Society of Chemistry 2021

SUPPORTING INFORMATION

Identification, Isolation, Structural Characterization, *In Silico* Toxicity Prediction and *In Vitro* Cytotoxicity Assay of Simeprevir Acidic and Oxidative Degradation Products

Rasha M. Ahmed[a], Marwa A.A. Fayed[b], Mohammed F. El-Behairy[c], Inas A. Abdallah[d#]

[a]Department of Pharmaceutical Chemistry, Faculty of Pharmacy, Misr International University, Cairo 11341, Egypt.

[b]Department of Pharmacognosy, Faculty of Pharmacy, University of Sadat City, Sadat City 32897, Egypt.

[c]Department of Organic and Medicinal Chemistry, Faculty of Pharmacy, University of Sadat City, Sadat City 32897, Egypt.

[d]Department of Analytical Chemistry, Faculty of Pharmacy, University of Sadat City, Sadat City 32897, Egypt.

# Corresponding author
Inas A. Abdallah
Department of Analytical Chemistry, Faculty of Pharmacy, University of Sadat City, Sadat City, Egypt
E-mail: inas.abdallah@fop.usc.edu.eg

Table of Contents

Figure S1 UV Spectrum of Simeprevir...S3

Figure S2 ^{1}H NMR Spectrum of Simeprevir...S4

Figure S3 ^{1}H NMR Spectrum of DP 1... S5

Figure S4 ^{1}H NMR Spectrum of DP 2... S6

Figure S5 ^{1}H NMR Spectrum of DP 3.. S7

Figure S6 ^{1}H NMR Spectrum of DP 4.. S8

Figure S7 ^{1}H NMR Spectrum of DP 5.. S9

Figure S8 Postulated mechanism of Simeprevir degradation to acidic degradation products …….S10

Figure S9 Postulated mechanism of Simeprevir degradation to oxidative degradation products …S11

Figure S10 Coefficient plots for method robustness ………………………………………….........S12

Figure S11 Optical microscope stained images of cytotoxicity assays at HSF cell line……………S13
(a)DP1, (b) DP2, (c) DP 3, (d) DP4, (e) DP5 and (f) Doxorubicin. All at concentration of 100 µM and Magnification power: 200x

Table S1 *In vitro* cytotoxicity assay of Simeprevir degradation products on HSF cell line………...S14

Figure S1- UV Spectrum of Simeprevir

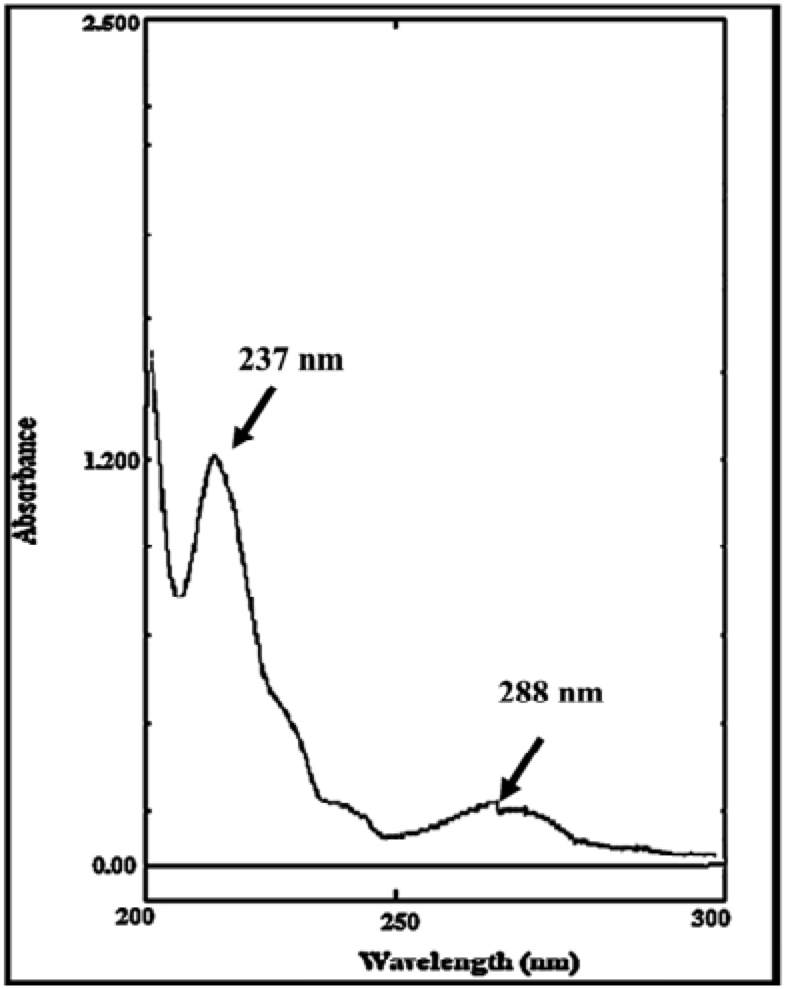

Figure S2-^{1}H NMR Spectrum of Simeprevir

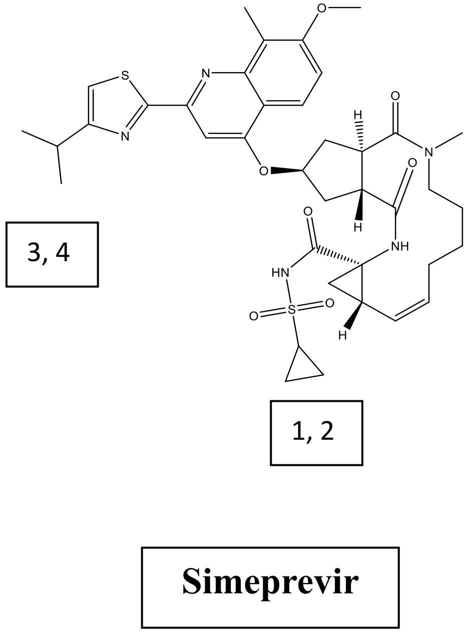

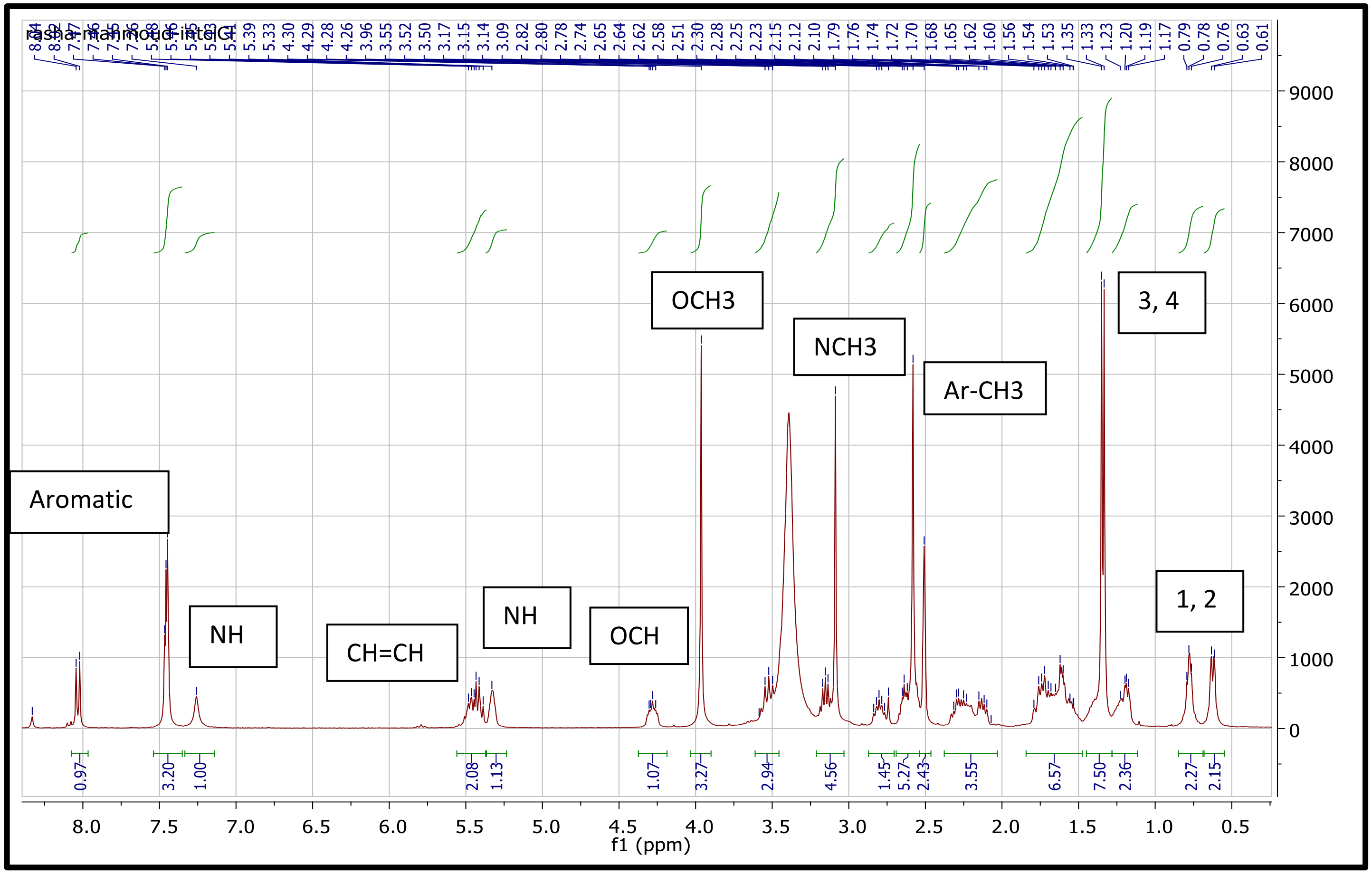

Figure S3-^{1}H NMR Spectrum of DP 1

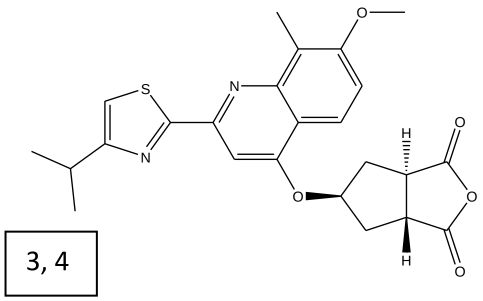

3, 4

DP 1

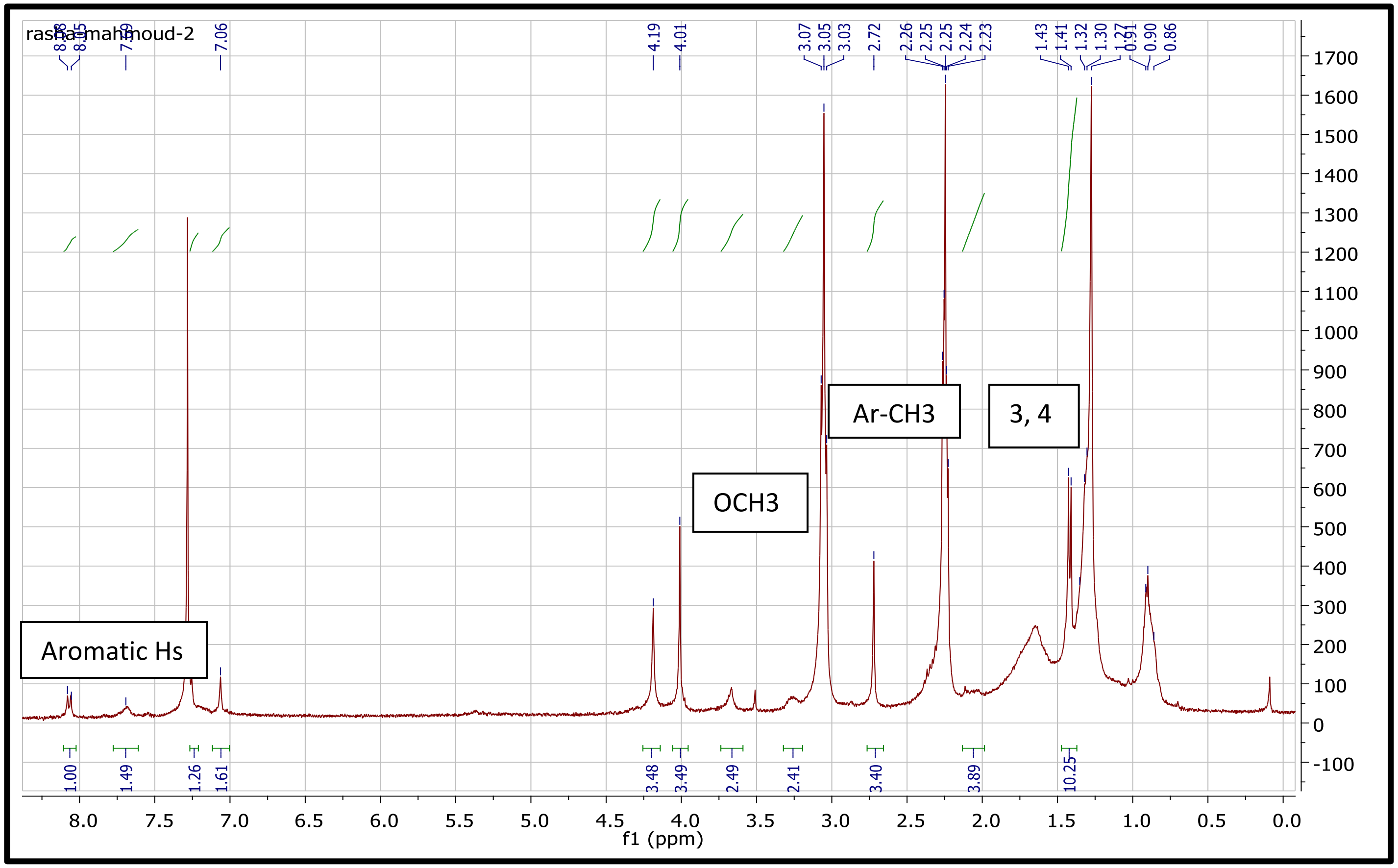

Figure S4-^{1}H NMR Spectrum of DP 2

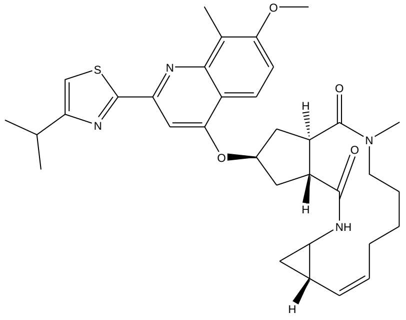

DP 2

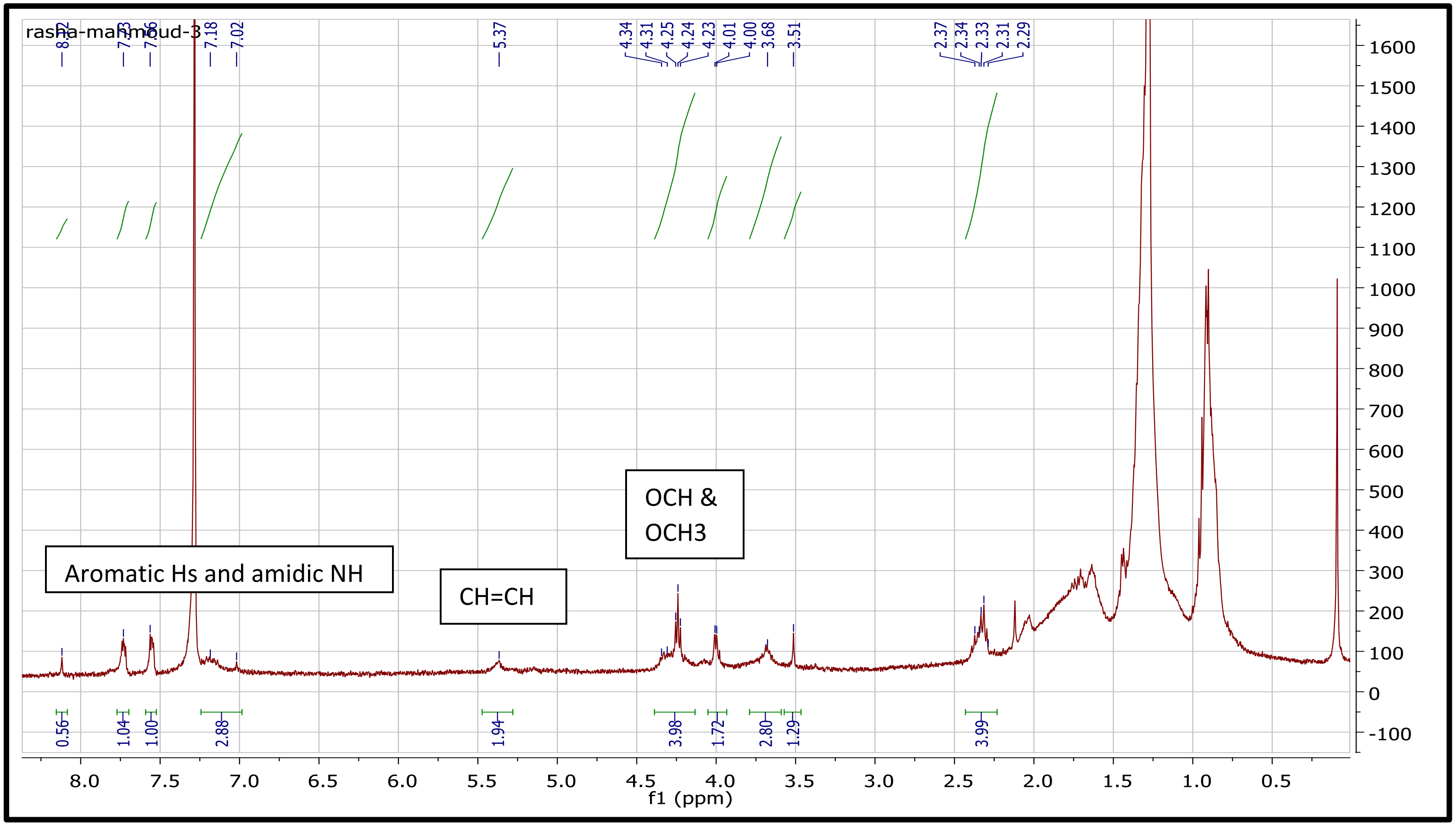

Figure S5-^{1}H NMR Spectrum of DP 3

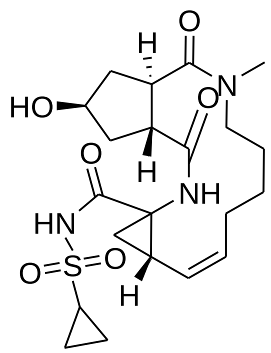

DP 3

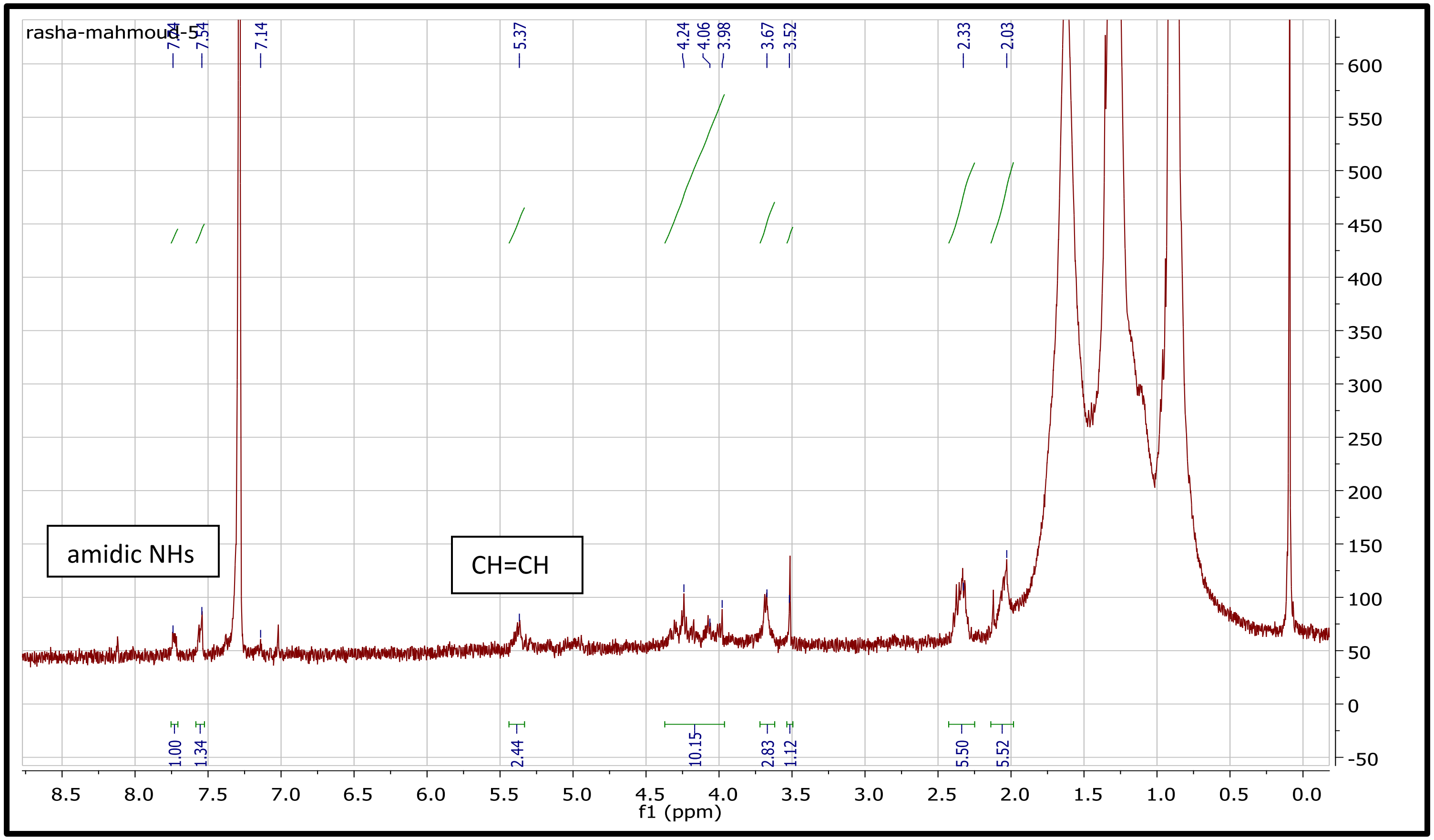

Figure S6-^{1}H NMR Spectrum of DP 4

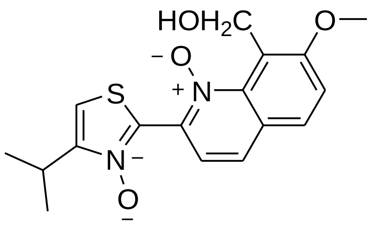

DP 4

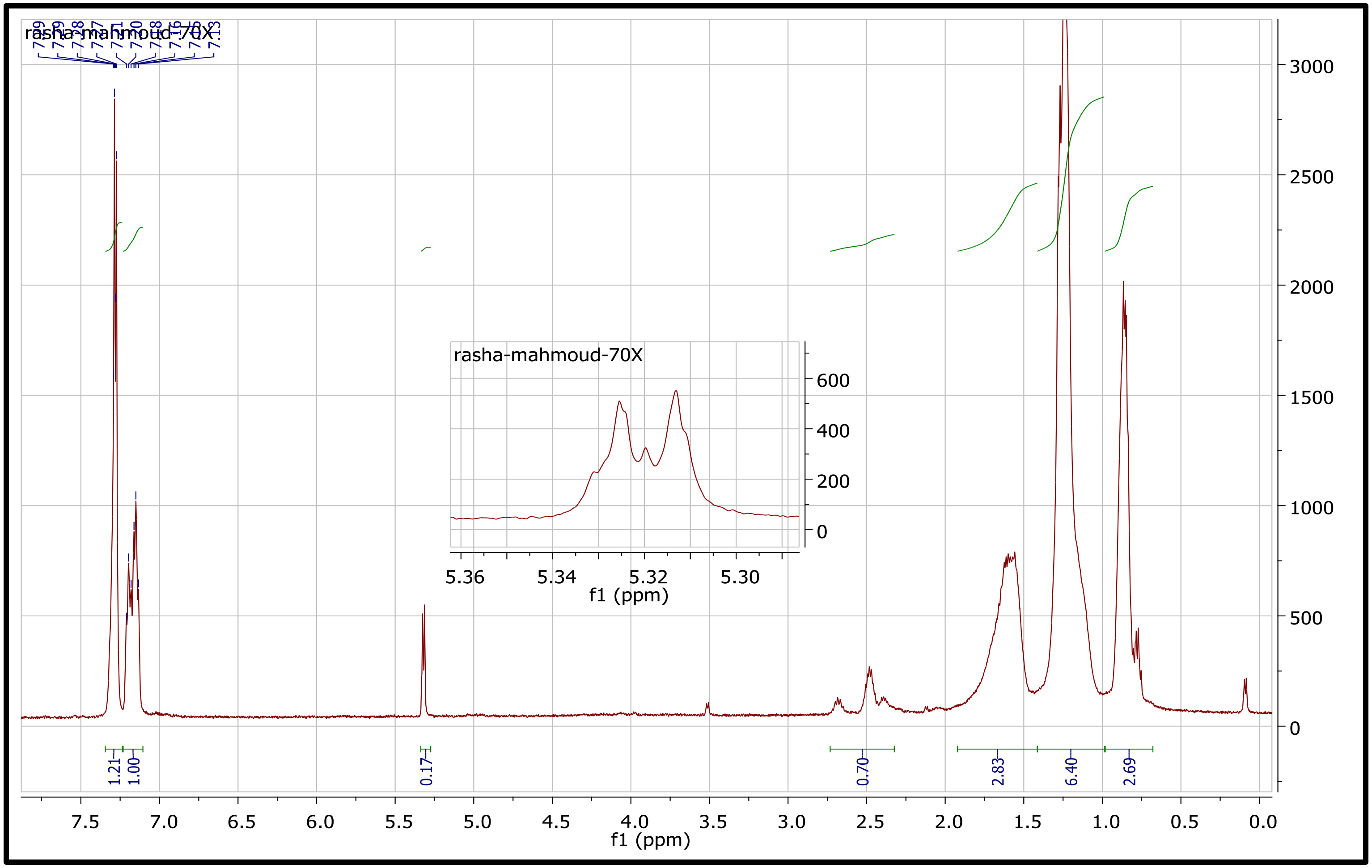

Figure S7-^{1}H NMR Spectrum of DP 5

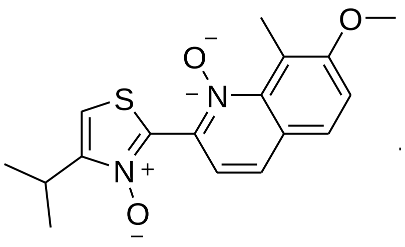

DP 5

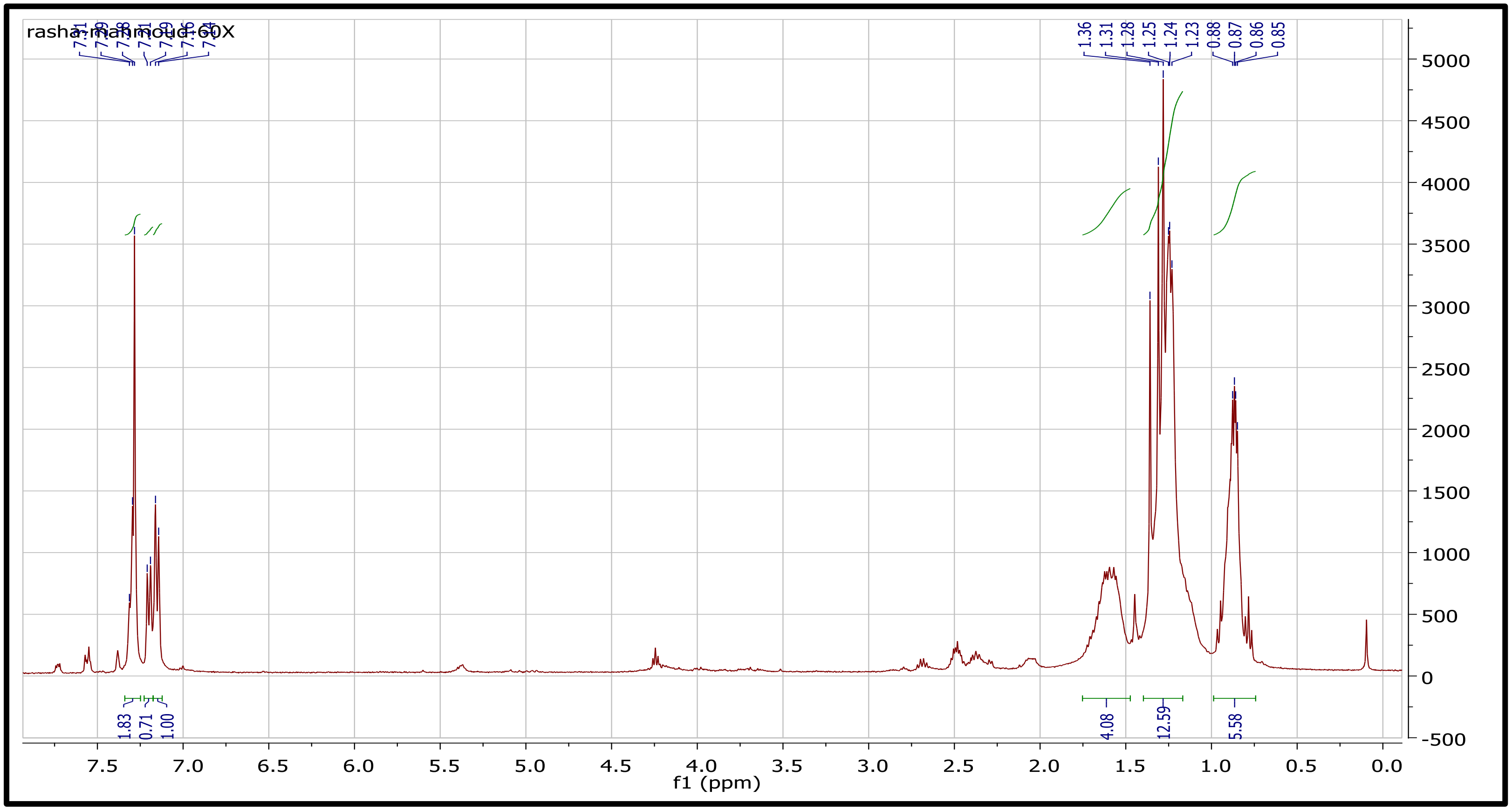

Figure S8- Postulated Mechanism of Simeprevir degradation to acidic degradation products

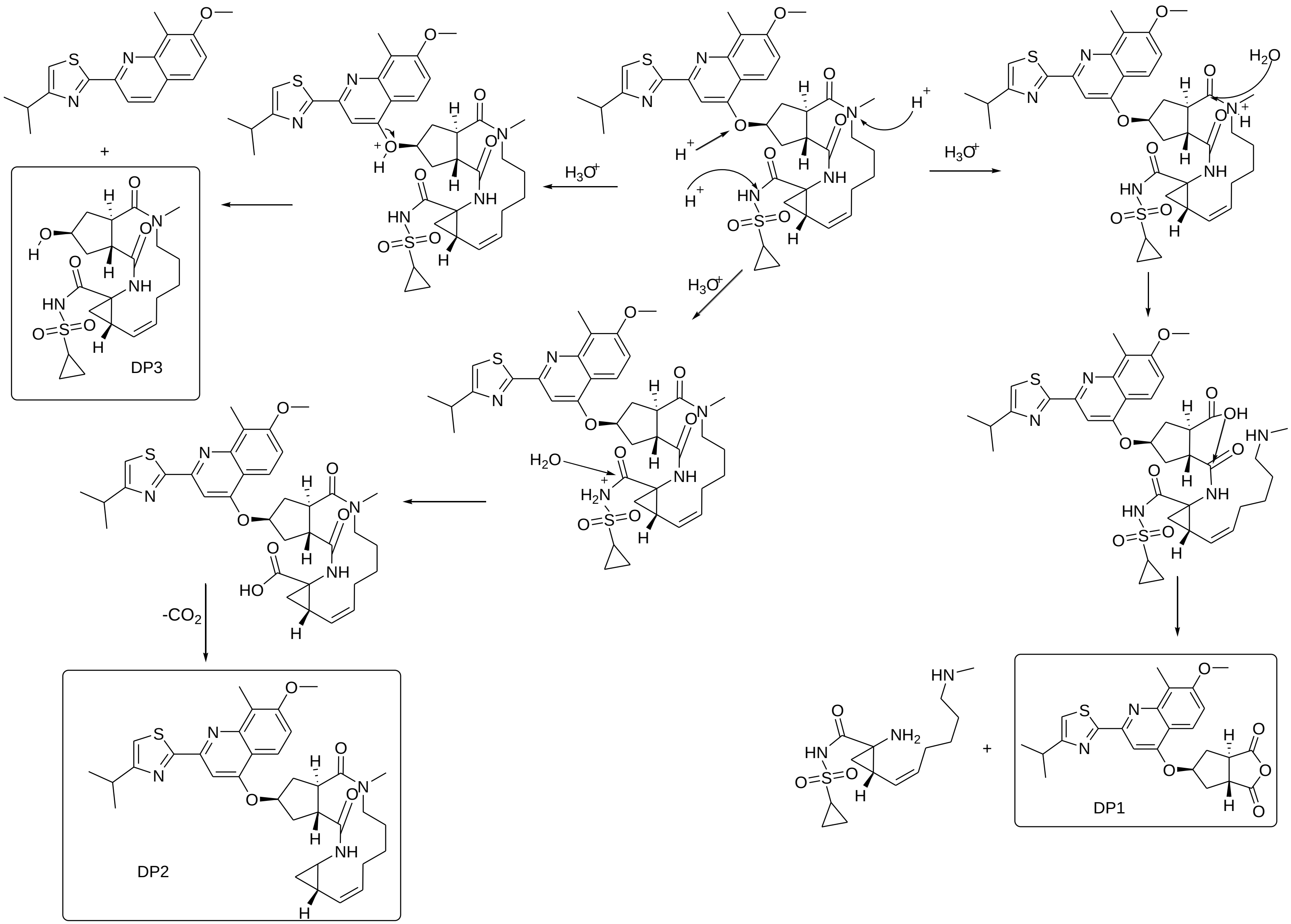

Figure S9- Postulated Mechanism of Simeprevir degradation to oxidative degradation products

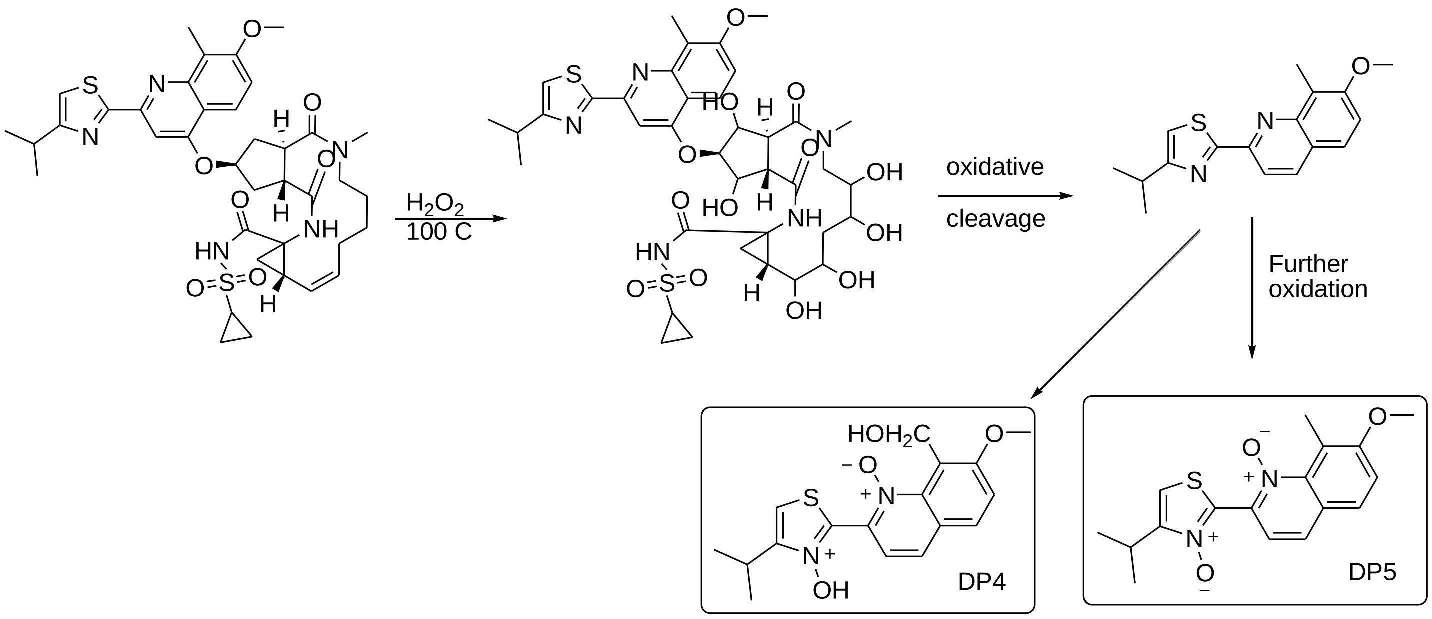

Figure S10- Coefficient plots for method robustness

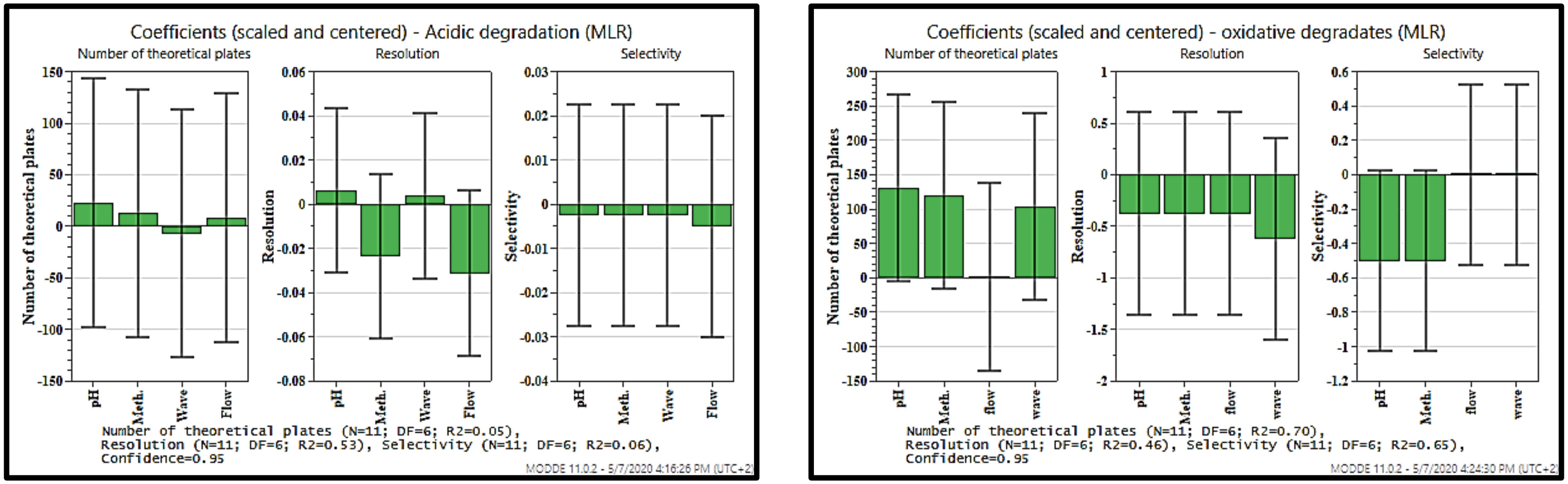

Figure S11- Optical microscope stained images of cytotoxicity assays at HSF cell line (a) DP1, (b) DP2, (c) DP 3, (d) DP4, (e) DP5 and (f) Doxorubicin. All at concentration of 0.1 µM and Magnification power: 200x

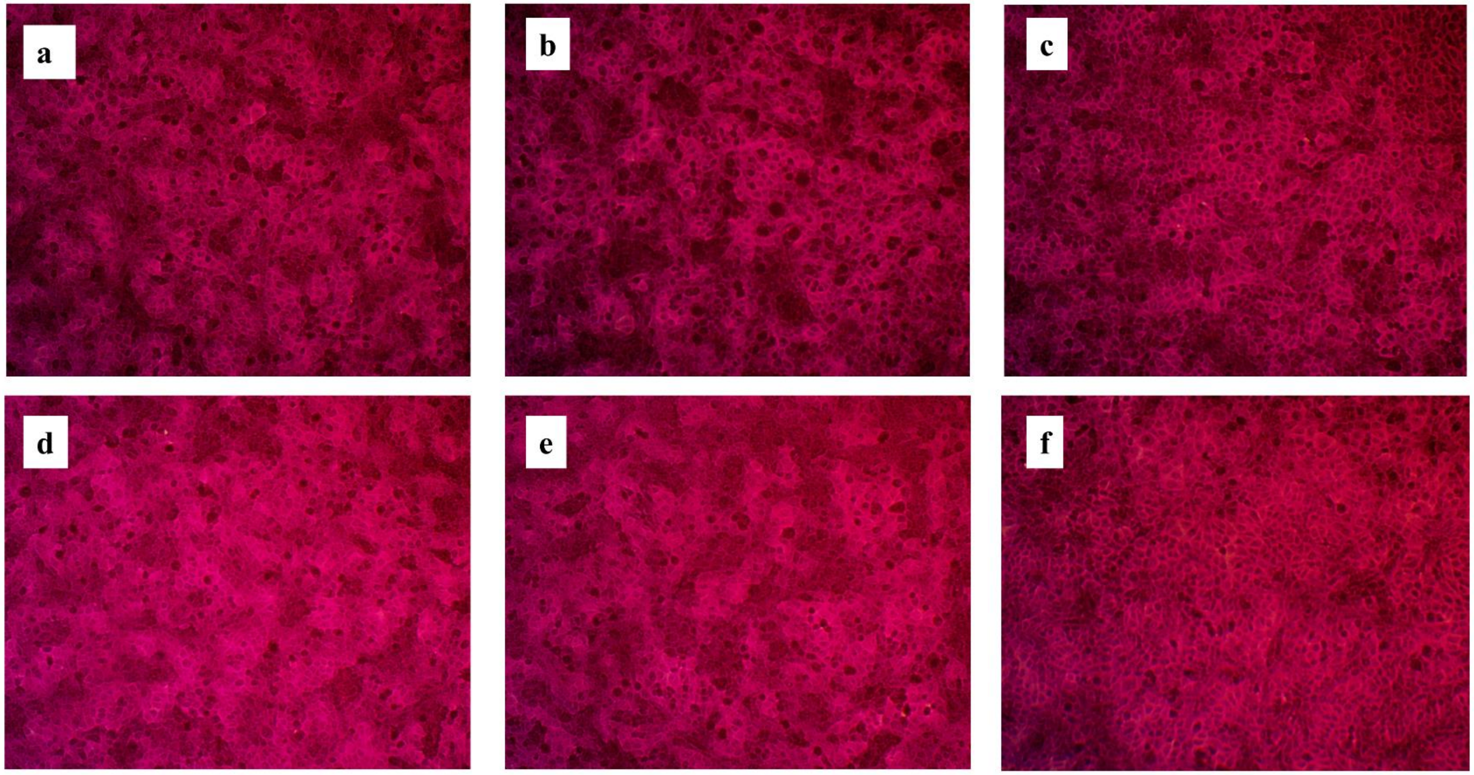

Table S1- In vitro cytotoxicity of simeprevir degradation products on Human Skin Fibroblast (HSF) cell line

DP1	Raw data			Blank Corrected Data			Viability %				
Conc.	1	2	3	1	2	3	1	2	3	Mean	STD
c	2.211	2.187	2.199	2.18	2.156	2.168	100	100	100	100	0
0.01	2.171	2.163	2.177	2.14	2.132	2.146	98.708	98.339	98.985	98.678	0.2645241
0.1	2.123	2.139	2.108	2.092	2.108	2.077	96.494	97.232	95.803	96.51	0.5838511
1	2.106	2.1	2.103	2.075	2.069	2.072	95.71	95.434	95.572	95.572	0.1129838
10	2.094	2.093	2.095	2.063	2.062	2.064	95.157	95.111	95.203	95.157	0.0376613
100	2.049	2.046	2.043	2.018	2.015	2.012	93.081	92.943	92.804	92.943	0.1129838
Blank	0.031	0.031	0.031	Blank Average		0.031	Control average		2.168		

DP2	Raw data			Blank Corrected Data			Viability %				
Conc.	1	2	3	1	2	3	1	2	3	Mean	STD
c	2.146	2.142	2.144	2.115	2.111	2.113	100	100	100	100	0
0.01	2.113	2.119	2.104	2.082	2.088	2.073	98.533	98.817	98.107	98.486	0.2917375
0.1	2.098	2.129	2.113	2.067	2.098	2.082	97.823	99.29	98.533	98.549	0.5990484
1	2.082	2.095	2.088	2.051	2.064	2.057	97.066	97.681	97.35	97.366	0.2514178
10	2.087	2.079	2.083	2.056	2.048	2.052	97.302	96.924	97.113	97.113	0.1545663
100	2.058	2.044	2.051	2.027	2.013	2.02	95.93	95.267	95.599	95.599	0.2704911
Blank	0.031	0.031	0.031	Blank Average		0.031	Control average		2.113		

DP3	Raw data			Blank Corrected Data			Viability %				
Conc.	1	2	3	1	2	3	1	2	3	Mean	STD
c	2.28	2.27	2.25	2.249	2.239	2.219	100	100	100	100	0
0.01	2.217	2.229	2.203	2.186	2.198	2.172	97.778	98.315	97.152	97.749	0.4752461
0.1	2.256	2.168	2.213	2.225	2.137	2.182	99.523	95.587	97.6	97.57	1.6070796
1	2.133	2.105	2.119	2.102	2.074	2.088	94.021	92.769	93.395	93.395	0.5112995
10	2.068	2.057	2.079	2.037	2.026	2.048	91.114	90.622	91.606	91.114	0.4017353
100	2.062	2.085	2.04	2.031	2.054	2.009	90.845	91.874	89.861	90.86	0.8217989
Blank	0.031	0.031	0.031	Blank Average		0.031	Control average		2.2357		

DP4	Raw data			Blank Corrected Data			Viability %				
Conc.	1	2	3	1	2	3	1	2	3	Mean	STD
**c**	2.2115	2.244	2.179	2.1805	2.213	2.148	100	100	100	100	0
**0.01**	2.126	2.12	2.132	2.095	2.089	2.101	96.079	95.804	96.354	96.079	0.2246723
**0.1**	2.078	2.044	2.112	2.047	2.013	2.081	93.878	92.318	95.437	93.878	1.273143
**1**	2.064	2.06	2.057	2.033	2.029	2.026	93.235	93.052	92.914	93.067	0.1315039
**10**	2.055	2.024	2.039	2.024	1.993	2.008	92.823	91.401	92.089	92.104	0.5805041
**100**	2.053	2.038	2.068	2.022	2.007	2.037	92.731	92.043	93.419	92.731	0.5616807
**Blank**	0.031	0.031	0.031	Blank Average		**0.031**	**Control average**		**2.1805**		

DP5	Raw data			Blank Corrected Data			Viability %				
Conc.	1	2	3	1	2	3	1	2	3	Mean	STD
**c**	2.19	2.228	2.266	2.159	2.197	2.235	100	100	100	100	0
**0.01**	2.152	2.147	2.142	2.121	2.116	2.111	96.541	96.313	96.086	96.313	0.1858208
**0.1**	2.128	2.187	2.157	2.097	2.156	2.126	95.448	98.134	96.768	96.783	1.0963952
**1**	2.125	2.054	2.089	2.094	2.023	2.058	95.312	92.08	93.673	93.688	1.3193713
**10**	2.042	2.105	2.073	2.011	2.074	2.042	91.534	94.401	92.945	92.96	1.1707202
**100**	2.049	2.078	2.063	2.018	2.047	2.032	91.853	93.173	92.49	92.505	0.5389871
**Blank**	0.031	0.031	0.031	Blank Average		**0.031**	**Control average**		**2.197**		

Dox	Raw data			Blank Corrected Data			Viability %				
Conc.	1	2	3	1	2	3	1	2	3	Mean	STD
**c**	1.954	1.97	1.955	1.923	1.939	1.924	100	100	100	100	0
**0.01**	1.955	1.95	1.944	1.924	1.919	1.913	99.758	99.499	99.188	99.482	0.2331617
**0.1**	1.928	1.931	1.928	1.897	1.9	1.897	98.358	98.514	98.358	98.41	0.073326
**1**	1.186	1.175	1.197	1.155	1.144	1.166	59.886	59.316	60.456	59.886	0.4656825
**10**	0.358	0.303	0.33	0.327	0.272	0.299	16.955	14.103	15.503	15.52	1.1642703
**100**	0.096	0.093	0.0945	0.065	0.062	0.0635	3.3702	3.2147	3.2924	3.2924	0.0635022
**Blank**	0.031	0.031	0.031	Blank Average		**0.031**	**Control average**		**1.9287**		
